# The worthy role of hepatic arterial infusion chemotherapy in combination with anti-programmed cell death protein 1 monoclonal antibody immunotherapy in advanced hepatocellular carcinoma

**DOI:** 10.3389/fimmu.2023.1284937

**Published:** 2023-10-31

**Authors:** Yixin Ding, Shasha Wang, Zhenkang Qiu, Chunyang Zhu, Yan Wang, Shufen Zhao, Wensheng Qiu, Kongjia Wang, Jing Lv, Weiwei Qi

**Affiliations:** ^1^ Department of Oncology, The Affiliated Hospital of Qingdao University, Qingdao University, Qingdao, China; ^2^ Interventional Medical Center, The Affiliated Hospital of Qingdao University, Qingdao, China; ^3^ Qingdao Municipal Hospital, Qingdao University, Qingdao, China

**Keywords:** HAIC, anti-PD-1, immunotherapy, HCC, combined therapy

## Abstract

Systemic therapy remains the primary therapeutic approach for advanced hepatocellular carcinoma (HCC). Nonetheless, its efficacy in achieving control of intrahepatic lesions is constrained. Hepatic arterial infusion chemotherapy (HAIC) is a therapeutic approach that combines localized treatment with systemic antitumor effects, which aim is to effectively manage the progression of cancerous lesions within the liver, particularly in patients with portal vein tumor thrombosis (PVTT). Combining HAIC with anti-programmed cell death protein 1 (anti-PD-1) monoclonal antibody (mAb) immunotherapy is anticipated to emerge as a novel therapeutic approach aimed at augmenting the response inside the localized tumor site and achieving prolonged survival advantages. In order to assess the effectiveness, safety, and applicability of various therapeutic modalities and to address potential molecular mechanisms underlying the efficacy of HAIC-sensitizing immunotherapy, we reviewed the literature about the combination of HAIC with anti-PD-1 mAb therapies.

## 1 Introduction

Primary liver cancer is a significant cause of death globally, occupying the third position among cancer-related mortalities. Hepatocellular carcinoma (HCC) is the predominant pathological form with the 5-year survival rate ranging from 12.1% to 19% (1–5). Around half of the patients who are diagnosed with advanced HCC are recommended to undergo systemic therapy (6). Currently, there is ongoing controversy among mainstream treatment guidelines worldwide over the optimal first-line treatment for advanced HCC. American and European liver disease guidelines recommend targeted drugs and anti-PD-1 mAb monoclonal antibody drugs as advanced HCC first-line treatment. Nevertheless, in the context of advanced HCC, a significant number of patients exhibit a substantial tumor burden and vascular invasion. Consequently, immunotherapy as a standalone systemic treatment option yields only modest improvements in overall survival. Hence, integrating local therapy and systemic therapy is progressively gaining acceptance (7–11).

Hepatic arterial infusion chemotherapy (HAIC) is an interventional therapy for the hepatic artery that technically resembles a local treatment but can have systemic therapeutic effects. Compared to direct intravenous chemotherapy, HAIC circumvents the first-pass effect of the liver, enhancing the efficacy of local treatment and decreasing the systemic toxicity of chemotherapy medications (12). In patients with advanced HCC with PVTT, the objective response rate (ORR) achieved through HAIC as a standalone treatment can range from 48% to 71%. A high ORR directly impacts the downgrading of tumors and the transformation of advanced unresectable HCC. Furthermore, patients fortunate to have surgery will experience improved long-term prognoses (13–15). The combination of HAIC and immunotherapy exhibits a positive impact on reducing tumor size in individuals with advanced HCC, leading to improved long-term survival outcomes. In this analysis, we examined the literature about HAIC and anti-PD-1 mAb combined therapy, and investigated the potential mechanisms by which HAIC may augment the effectiveness of immunotherapy in the heterogeneous and suppressive immune microenvironment of HCC.

## 2 Regimens and technique of HAIC

Current chemotherapeutic agents now employed for HAIC primarily consist of one or more cytotoxic chemotherapeutic agents, such as platinums, 5-Fluorouracil (5-Fu), gemcitabine, doxorubicin, and epirubicin. i) Epirubicin-HAIC: This regimen was the initial chemotherapeutic approach employed in the clinical setting. However, this regimen is associated with certain limitations in terms of its efficacy and the occurrence of significant side effects. Consequently, its utilization has become infrequent in contemporary clinical practice (16, 17). ii) GEMOX-HAIC: This regimen is predominantly utilized in France and other European nations (18–20). The GEMOX regimen exhibits lower nephrotoxicity and hepatotoxicity compared to the cisplatin regimen, which will be discussed next. This characteristic renders this treatment protocol more appropriate for those diagnosed with HCC accompanied by cirrhosis. However, this regimen is prone to thrombocytopenic AEs, which may cause gastrointestinal hemorrhage in patients with concomitant esophagogastric fundal varices (21). iii) CDDP-HAIC or FP-HAIC: Cisplatin-based solutions are mostly used in Japan and Korea. Multiple studies have conclusively demonstrated the fundamental significance of HAIC regimens featuring cisplatin (CDDP) as the primary chemotherapeutic agent (13, 22–24) FP-HAIC is the most common solution (13, 25, 26). Cisplatin monotherapy treats about 11% of the population and shows superior efficiency and outcome than targeted therapy (27–32). iv) FOLFOX-HAIC: FOLFOX-HAIC regimen was first proposed by Chinese scholars (33), and the survival improvement of FOLFOX-HAIC compared with sorafenib monotherapy was confirmed in phase I/II or retrospective studies (34–39).

Currently, there exist two distinct techniques for delivering HAIC (40–42):i) Continuous perfusion chemotherapy with disposable catheterization: The Seldinger method is employed for the purpose of puncturing the right femoral artery or other arteries. Subsequently, a catheter is inserted into the punctured artery, allowing for angiography to be performed in the abdominal cavity, explicitly targeting the upper and superior mesenteric arteries. The microcatheter is inserted into the principal artery that supplies blood to the tumor. After the microcatheter placement, the visible segment of the catheter is affixed to the skin in the right femoral groin and lower abdomen. Subsequently, the administration of chemotherapeutic agents are carried out following the prescribed chemotherapy schedule, after which both the catheter and microcatheter are extracted. Angiography, cannulation, and fixation are recanalized before each HAIC. Every time the blood supply to the tumor changes, the blood supply vessel to the tumor should be recanalized. ii) Hepatic Arterial Chemotherapy Kit Continuous Perfusion Chemotherapy: The superior mesenteric artery and celiac artery are imaged when the femoral or subclavian artery is pierced. The catheter’s other end is attached to the cartridge, buried in the inguinal and subclavian areas, and left in the innominate hepatic artery. After hepatic arteriography revealed that the cartridge is in good working condition, continuous HAIC was carried out. While the latter helps prevent repeated arterial punctures, its hazards include thrombosis, infection, catheter migration, and the inability to alter the catheter location in response to changes in the tumor’s blood supply (26). Most FOLFOX-HAIC implementations use a single puncture placement strategy, which lowers the frequency of catheter-related adverse events while still guaranteeing the precision of the perfusion target area (36, 37).

## 3 Efficacy of combination therapy

### 3.1 HAIC plus targeted therapy

Sorafenib has significantly transformed the therapy approach for HCC (43–47) and has been used most frequently in studies on HAIC combination therapy. Administration of lenvatinib reduces angiogenesis, causes apoptosis, and enhances sensitivity to FOLFOX medications. Researches on lenvatinib combined with HAIC also showed the superior clinical outcome and tolerable AEs of combination therapy (48, 49). To better demonstrate following triple therapy, we provide thorough information regarding studies employing HAIC coupled with sorafenib or lenvatinib in **Table 1**.

**Table 1:** Retrospective and phase I/II clinical trials evaluating HAIC and targeted agent combinations in advanced HCC.

Agent	Trial description	Trial design	DCR/ORR (%)	mPFS/mOS (months)	Safety profile	Reference
**HAIC+Sorafenib**	**Phase I trial** **N=15**	CDDP-HAIC (CDDP: 30 mg/m^2^) + Sorafenib	RECIST 1.1 DCR: 73.3 ORR: 26.7	mPFS: 5.0 mOS: 9.7	NA	(31)
	**Phase II trial** **N=38**	CDDP-HAIC + Sorafenib	RECIST 1.1 DCR: 76.3 ORR: 57.9	mPFS: 6.2 mOS: 14.2	Grade 3-4 AEs: thrombocytopenia (13.2%), AST elevation (7.9%), amylase and bilirubin elevation (2.6%), creatinine increased (2.6%), hepatic encephalopathy (2.6%), allergic reaction (2.6%)	(28)
	**Retrospective** **Study** **N=71**	HAIC (Cisplatin, Mitomycin, 5-Fu and Leucovorin) + Sorafenib vs Sorafenib	mRECTST DCR: 88 vs 48 ORR: 73 vs 32	mPFS: 6 vs 4 ^a^ mOS: 12 vs 12^a^	NA	(32)
	**Phase III trial** **N=205**	FP-HAIC (Cisplatin: 20 mg/m², d1,8 +Fluorouracil: 330 mg/m² d1–5, d8–12, q4w) + Sorafenib vs Sorafenib	RECIST 1.1 ORR: 36 vs 18	mTTP: 5.3 vs 3.5 mPFS: 4.8 vs 3.5 ^a^ mOS: 11.8 vs 11.5 ^a^	All AEs were similar in two groups. Grade 3-4 AEs: anemia (17% vs 6%), neutropenia (17% vs 1%), thrombocytopenia (34% vs 12%), anorexia (14% vs 6%). More frequent in sorafenib group: alopecia, hoarseness, diarrhea and elevated alanine aminotransferase. More frequent in combination group: nausea, vomiting and decreased white cell count.	(26)
	**Phase II trial** **N=106**	CDDP-HAIC (65 mg/m^2^, q4-6w) + Sorafenib vs Sorafenib	mRECIST Response rate: 21.7 vs 7.3^a^	mTTP: 3.1 vs 2.8 ^a^ mOS: 10.6 vs 8.7 ^a^	More frequent in combination group: neutropenia, leukopenia, thrombocytopenia, hyponatremia, nausea and hiccups.	(29)
	**Phase I trial** **N=18**	Interval FP-HAIC (CDDP: 20 mg/m^2^ d1, 8 + 5-Fu: 330 mg/m^2^ d1-5, d8-12, q3w) + Sorafenib	RECIST 1.1 DCR: 77.8 ORR: 38.9	mTTP: 9.7 mOS: 14.1	All AEs except hematology: hand-foot skin reactions (61.1%), diarrhea (33.3%), appetite loss (27.8%), hypertension (22.2%); Grade 3 hematology AEs: leucopenia (16.7%), neutropenia (11.1%), anemia (11.1%), thrombocytopenia (16.7%)	(25)
	**Retrospective** **Study** **N-98**	Long-interval FP-HAIC (treatment cycle 2-3 m) + Sorafenib	mRECIST DCR; 69.2 vs 44.4 ORR: 23.1 vs. 6.9 ^a^	mOS: 17.1 vs 9.7	Grade 3-4 AEs were similar in two groups.	(50)
	**Phase II trial** **N=83**	GEMOX-HAIC (Gemcitabine: 1000 mg/m^2^, d1 + Oxaliplatin: 100 mg/m^2^, d2, q4w) + Sorafenib vs Sorafenib	RECIST DCR: 77 vs 71 ORR: 15 vs 9 mRECIST ORR: 28.2 vs 20.5	mPFS: 6.2 vs 4.6 mOS: 13.5 vs 14.8	Grade 3-4 AEs: neutropenia (23% vs 0%), fatigue (21% vs 7%), thrombocytopenia (33% vs 0%), diarrhea (18% vs 9%), peripheral neuropathy (5% vs 0%), hand–foot syndrome (5% vs 18%).	(18)
	**Phase II trial**	FOLFOX-HAIC (Oxaliplatin, 85 mg/m^2^, d1; Leucovorin, 400mg/m^2^, d1; 5-Fu, 400mg/m^2^, bolus at d1 and 2400 mg/m^2^ over 46 h, d1-2) + Sorafenib	RECIST DCR: 77.1 ORR:40 mRECIST DCR: 77.0 ORR:62.8	mPFS: 6.7 mOS: 13.2	Grade 3-4 AEs: AST elevation (31.4%), hand-foot syndrome (17.1%), thrombocytopenia (14.3%), neutropenia (8.6%), esophageal hemorrhage (8.6%).	(36)
	**Phase III trial** **N=245**	FOLFOX-HAIC + Sorafenib	RECIST ORR; 40.8 vs 2.46 DCR: 75.2 vs 49.2 mRECIST ORR; 54.4 vs 7 DCR: 76.0 vs 50.8	mPFS: 7.03 vs 2.6 mOS: 13.37 vs 7.13	All AEs were similar in two groups. Grade 3-4 AEs: neutropenia (9.6% vs 2.48%), thrombocytopenia (12.9% vs 4.96%), vomiting (6.45% vs 0.83%).	(37)
	**Retrospective study** **N=225**	FOLFOX-HAIC + Sorafenib vs FOLFOX-HAIC	RECIST 1.1 DCR: 74.2 vs 55.9 ORR; 36.6 vs 33.3	mPFS: 6.9 vs 4.1 mOS: 13.0 vs 10.0	Grade 3–4 hand–foot skin reaction was more frequent in the combination group	(39)
	**Phase II trial** **N=39**	Long-interval FOLFOX-HAIC + Sorafenib	RECIST 1.1 ORR: 18 DCR: 69 mRECIST ORR: 28	mTTP: 7.7 mOS: 15.1	Grade 1-2 AEs: fatigue (69%), hypophosphatemia (64%), diarrhea (59%), ALT elevation (56%), nausea (54%), thrombocytopenia (46%). Grade 3 AEs: AST elevation (28%), ALT elevation (15%), diarrhea (13%), bilirubin (10%), anemia (10%), hand-foot syndrome (8%), bleeding (8%).	(51)
	**Phase II trial** **N=64**	FOLFOX-HAIC + Sorafenib vs Sorafenib	RECIST 1.1 ORR: 41 vs 3 mRECIST ORR: 50 vs 3	mPFS: 9.0 vs 2.5 mOS: 16.3 vs 6.5	All AEs were similar in two groups. (91% vs 88%) Grade 3-4 AEs: diarrhea (22% vs 16%), hand-foot syndrome (19% vs 6%), thrombocytopenia (22% vs 0%), and AST elevation (6% vs 3%)	(52)
**HAIC+Lenvatinib**	**Retrospective study** **N=150**	FOLFOX-HAIC+ Lenvatinib vs FOLFOX-HAIC+ Lenvatinib+ Microwave ablation	NA	mPFS: 5.6 vs 12.8 mOS: 13.6 vs >30	All AEs were similar in two groups.	(48)
	**Retrospective study** **N=242**	FOLFOX-HAIC+ Lenvatinib vs FOLFOX-HAIC	ALBI score ^b^: -2.60 to -2.34 vs -2.56 to -2.34 ^a^	mPFS: 19.4 vs 16.2 ^a^ 3-year cumulative OS rates: 63.6, 12.1, 3.0 vs 47.2, 11.8, 2.7	All AEs were similar in two groups. Grade 3-4 AEs: leukopenia (0.8%), neutropenia (0.8%), vomiting (1.5%), and ALT elevation (1.5%)	(49)

AEs, adverse effects; mPFS, median progression-free survival; mOS, median overall survival; 1-year OS, 1-year overall survival rate; ORR, objective response rate; DCR, disease control rate; mTTP, median time to progression; ALBI, albumin-bilirubin; BI, total bilirubin; AL, albumin. NA, not available

^a^: There was no significant statistically difference between the two groups.

^b^: AALBI score (53) = (log 10 BI (μmol/L) × 0.66) + (AL (g/L) × − 0.085), (grade 3, >− 1.39; grade 2, − 2.60 to − 1.39; grade 1, ≤ − 2.60).

### 3.2 HAIC plus anti-PD-1 mAb

#### 3.2.1 Dual therapy

Large-scale, randomised, controlled studies are currently lacking for the dual combination of HAIC and immunotherapy. Existing retrospective studies have demonstrated a greater disease control rate (DCR) and survival benefit when FOLFOX-HAIC is paired with anti-PD-1 mAb monoclonal antibody medications. Nonetheless, no substantial disparity in ORR was observed between the two cohorts when assessed using the modified Response Evaluation Criteria in Solid Tumors (mRECIST). Furthermore, the group receiving combination therapy did not achieve any survival advantage in patients with PVTT or extrahepatic metastases (54). According to a second retrospective research, HAIC (Epirubicin + CDDP) combined with ICI had a much better vascular response than any single regimen therapies. However, there was no discernible difference in the three groups’ survival rates, presumably due to the fact that 85.4% of the individuals in this experiment were diagnosed with PVTT Vp3/4 (54, 55). Moreover, toripalimab combined with FOLFOX-HAIC demonstrated superior efficacy in terms of survival benefit compared to lenvatinib monotherapy. Despite the exclusion of individuals with advanced HCC and centra nervous system (CNS) metastases, a considerable survival benefit and manageable treatment-related adverse events (AEs) were achieved (56).

#### 3.2.2 Triple therapy

The immune checkpoint inhibitor atelizumab is a humanized anti-PD-L1 mAb that blockade the combination of PD-1 and B7. Atelizumab exhibits immunological activity against tumor cells by restoring the tumor cell killing effect of T cells (57). Bevacizumab is an antibody that exhibits anti-angiogenic properties and modulates the immune system that enhances anti-cancer immunity (58). The coadministration of the atelizumab (trade name: Tecentriq) and bevacizumab (trade name: Avastin) is commonly denoted as “T+A”. The National Comprehensive Cancer Network (NCCN) recommended the use of the “T+A” regimen as the initial treatment for patients with unresectable advanced HCC. This regimen demonstrated an ORR of 25.1%, thereby establishing a basis for the application of targeted therapy in combination with anti-PD-1 mAb treatment (59, 60). Triple therapy is used for rapid tumor shrinkage and disease control, followed by targeted immunological combination maintenance therapy for continuous tumor control, which has the potential to impede disease advancement and extend patient survival considerably. The paradigm under consideration is established to address HCC by emulating the principles of conventional chemotherapy. Compared with dual therapy combined with targeted immunity, triple induction therapy combined with HAIC showed superior efficacy in terms of survival outcomes and tumor control of intrahepatic and extrahepatic lesions. However, due to the relatively poor economic conditions of Chinese HCC patients, most studies chose toripalimab in triple combination therapy instead of nivolumab and pembrolizumab.

The specific situation of triple therapy based on lenvatinib and toripalimab displayed superior tumor response and survival outcome than monotherapy or targeted plus anti-PD-1 mAb therapy (56). Most studies of triple therapy include multiple immune drugs (toripalimab, pembrolizumab, nivolumab and sintilimab, etc.), given the influence of factors like socioeconomics on the selection of immunotherapy drugs and the lack of significant randomized controlled studies that strictly control variables. The findings demonstrated that in terms of treatment response and survival advantages, the triple therapy group outperformed the target-immune dual therapy group (54, 61–63). In addition, triple therapy also delayed extrahepatic tumor progression compared with dual therapy (61). In contrast to other solid tumors, immunotherapy in advanced HCC does not necessitate particular criteria for the expression level of PD-L1. Nevertheless, research findings have indicated a positive correlation between increased tumor mutation burden (TMB), as measured by combined positive score (CPS), and the efficacy of pembrolizumab in stimulating the immune response. Consequently, patients with a higher CPS are more likely to derive therapeutic benefits from pembrolizumab (62).

The superiority of triple therapy has also been demonstrated in other retrospective studies that included multiple tyrosine kinase inhibitor (TKI)-targeted agents (lenvatinib, regorafenib, sorafenib, and apatinib) (64, 65). In addition to TKI drugs, the triple therapy regimen incorporated an anti-vascular endothelial growth factor receptor (VEGFR) agent known as bevacizumab, resulting in notable tumor response (66). Additional information on HAIC and anti-PD-1 mAb combined therapy was included in **Table 2**.

**Table 2:** Retrospective and phase I/II clinical trials evaluating HAIC, anti-PD-1 mAb, and/or targeted agent combinations in advanced HCC.

Agent	Trial description	Trial design	DCR/ORR (%)	mPFS/mOS (months)	Safety profile	Reference
**HAIC + Anti-PD-1 mAb**	**Retrospective study** **N=229**	FOLFOX-HAIC + Anti-PD-1 mAb^b^ vs HAIC	mRECIST ORR: 38 vs 30 ^a^ DCR: 83 vs 66 Intrahepatic response: 85 vs 74	mPFS: 10.0 vs 5.6 mOS: 18.0 vs 14.6	The most common AEs were pain, fever and vomiting.	(54)
**HAIC + Lenvatinib + Anti-PD-1 mAb**	**Retrospective study** **N=157**	FOLFOX-HAIC+ Lenvatinib + Toripalimab vs Lenvatinib	RECIST 1.1 ORR: 59.2 vs 9.3 DCR: 90.1 vs 72.1 mRECIST ORR: 67.6 vs 16.3 DCR: 90.1 vs 72.1	mPFS: 11.1 vs 5.1 mOS: NR vs 11	Grade 3–4 AEs: neutropenia (8.5% vs 1.2%), thrombocytopenia (5.6% vs 0%), nausea (5.6% vs 0%). Any grade liver dysfunction (elevated ALT, elevated AST, hyperbilirubinemia, hypoalbuminemia) was more frequent in combination group.	(56)
	**Phase II trial** **N=36**	FOLFOX-HAIC+ Lenvatinib + Toripalimab	RECIST 1.1 ORR: 63.9 DCR: 88.9 mRECIST ORR: 66.7 DCR: 88.9	mPFS: 10.9 mOS: 17.9	Grade 3–4 AEs: thrombocytopenia (13.9%), elevated AST (13.9%), and hypertension (11.1%). irAE: dermatitis (22.2%) and hypothyroidism (13.9%).	(67)
	**Retrospective study** **N=61**	FOLFOX-HAIC+ Lenvatinib + Anti-PD-1 mAb^c^	RECIST 1.1 ORR: 36.1 DCR: 82.0 mRECIST ORR: 57.4 DCR: 82.0	mPFS: 6.0 mOS: NA	Grade 3–4 AEs: abdominal pain (8.2%), neutropenia (6.6%), thrombocytopenia (4.9%), elevated ALT (3.3), elevated AST (3.3%).	(63)
	**Retrospective study** **N=142**	FOLFOX-HAIC+ Lenvatinib + Anti-PD-1 mAb^d^ vs Lenvatinib + Anti-PD-1 mAb^d^	mRECIST ORR: 61.8 vs 20.8 DCR: 86.5 vs 56.6	mPFS: 11.1 vs 5.5 mOS: 26.3 vs 13.8	Grade 3–4 AEs: platelet count decreases (21.3% vs. 7.5%) and elevated AST (36.0% vs. 9.4%).	(61)
	**Retrospective study** **N=170**	FOLFOX-HAIC+ Lenvatinib + Pembrolizumab vs Lenvatinib + Pembrolizumab	RECIST 1.1 ORR: 46.4 vs 30.2 DCR: 90.5 vs 83.8 mRECIST ORR: 59.5 vs 41.9 DCR: 88.1 vs 82.6	mPFS: 10.9 vs 6.8 mOS: 17.7 vs 12.6	All AEs were similar in two groups. Grade 3–4 AEs: 4.8% vs 2.3%.	(62)
**HAIC+ Targeted agent + Anti-PD-1 mAb**	**Retrospective study** **N=27**	FOLFOX-HAIC+ TKI^e^ + Anti-PD-1 mAb^f^	mRECIST ORR: 63.0 DCR: 92.6	mPFS: 10.6 (patients who had not previously received treatment: NR) mOS: NR	Grade 3 AEs: 55.6% Grade 1–2 AEs: thrombocytopenia (33.3%), elevated AST (44.4%), elevated total bilirubin (51.9%). Grade 1–2 irAE: hypothyroidism (29.6%), elevated serum amylase 1 (3.7%), elevated lipase (3.7%)	(64)
	**Retrospective study** **N=30**	FOLFOX-HAIC+ Lipiodol embolization + Targeted agent^g^ + Anti-PD-1 mAb^h^	RECIST 1.1 ORR: 63.3 DCR: 100 mRECIST ORR: 83.3 DCR: 100	mDOR: 10.3 mPFS: 13.2 1-year OS: 96.7	Grade 3 AEs: elevated serum bilirubin (3.3%), gastrointestinal bleeding (6.7%), stomachache (3.3%).	(65)
	**Retrospective study** **N=135**	FOLFOX-HAIC+ Targeted agent^i^ + Anti-PD-1 mAb^j^	mRECIST ORR: 54.1 DCR: 94.6	successful conversion surgery vs unsuccessful conversion surgery mPFS: 28 vs 7 mOS: 30 vs 15	Grade 3 AEs: fatigue, pain and fever.	(66)

NR, not reached; NA, not available; irAE, immune-related AEs; TKI, tyrosine kinase inhibitors; mDOR, median duration of response.

^a^: There was no significant statistically difference between the two groups.

^b^: Nivolumab (4%), Keytruda (5%), Toripalimab (60%), Sintilimab (35%);

^c^: Pembrolizumab (1.6%), Camrelizumab (60.7%), Tislelizumab (19.7%), Sintilimab (14.8%), Toripalimab (3.3%);

^d^: Pembrolizumab (11.2% vs 37.7%), Sintilimab (36.0% vs 26.4%), Toripalimab (40.4% vs 17.0%), Camrelizumab (6.7% vs 11.3%), Tislelizumab (5.6% vs 7.5%);

^e^: Sorafenib (18.5%), Lenvatinib (40.7%), Regorafenib (37.0%), Apatinib (3.7%);

^f^: Camrelizumab (66.7%), Sintilimab (26.1%), Toripalimab (7.4%), Nivolumab (7.4%);

^g^: Bevacizumab (3.3%), Lenvatinib (46.7%), Sorafenib (50.0%);

^h^: Sintilimab (40.0%), Carrelizumab (56.7%), Atezolizumab (3.3%);

^i^: Bevacizumab (42.2%), Lenvatinib (49.6%), Sorafenib (4.44%), Apatinib (3.70%);

^j^: Sintilimab (71.1%), Atezolizumab (0.74%), Camrelizumab (23.0%), Pembrolizumab (0.74%).

## 4 Safety and adverse effects of combination therapy

The severity of all AEs was evaluated to be minimal, requiring simple management. Furthermore, most investigations did not reveal any statistically significant disparities between the combination group and other groups. However, it is worth noting that the combined therapy utilizing HAIC is more prone to inducing chemotherapy-associated AEs. The safety profile shown in **Tables 1**, **2** suggested that the most common AEs were gastrointestinal AEs (diarrhea, nausea, vomiting, anorexia, and oxaliplatin-related abdominal pain), hematologic AEs (thrombocytopenia, neutropenia, leukopenia and anemia), rash, alanine aminotransferase (ALT) and/or aspartate aminotransferase (AST) elevated, hand-foot skin reaction, fever, hypertension and hypothyroidism in combined therapy. In particular, there was a higher prevalence of grade 3-4 AEs in the domains of abdominal pain, hematologic AEs, and elevated ALT and/or AST levels, likely attributable to their increased incidence. Moreover, higher frequency of hematologic AEs (especially neutropenia and thrombocytopenia), ALT and/or AST elevated, nausea, and vomiting were discovered in HAIC-combined therapy compared to targeted and/or anti-PD-1 mAb therapy. Nevertheless, it’s worth noting that the elevated transaminases caused by combined therapy were likely to return to normal soon after treatment (68), and there was no observed increase in immune-associated AEs (such as rash, hypothyroidism, hyperthyroidism, hypophysitis, pneumonitis and hepatitis) when comparing to the anti-PD-1 mAb monotherapy (67). In conclusion, investigating of the dosage and drug schedule of the HAIC regimen remains a valuable pursuit, as it has the potential to yield improved survival outcomes and reduced toxicity. Long-interval or low-dose with consecutive HAIC treatment may improve the synergy of chemotherapy drugs and provide lower treatment-related AEs and better survival outcome (25, 50–52, 69, 70).

In addition, various HAIC procedures impact the occurrence of AEs. The utilization of repeated catheterization and digital subtraction angiography (DSA) prior to each HAIC treatment cycle has exhibited superior dependability in the targeted delivery, as compared to implantable port catheter systems. According to a previous report, the occurrence of grade 3–4 AEs (e.g., catheter tip dislocation, thrombosis, and port-related infection) associated with implantable port catheter systems was <12% (71). Whether the difference in HAIC technology leads to differences in survival outcomes still needs to be confirmed by more prospective studies.

## 5 Molecular mechanisms of superior efficacy

The above studies have found that the FOLFOX-HAIC combination therapy is more beneficial than the CDDP-based HAIC combination therapy. Compared with cisplatin’s mechanism of inducing DNA damage, oxaliplatin promotes tumor cell death by inducing ribosome biosynthesis stress (72). Second, studies in colon cancer cells have shown that oxaliplatin can stimulate cancer cells to expose proapoptotic calreticulin (CRT), which is required for immunogenic cell death (ICD), and advocate for the effectiveness of anti-tumor therapy (73). Furthermore, the utilisation of oxaliplatin to provide HAIC demonstrates significant pharmacokinetic advantages (74). In addition, 5-Fu modulates the expression of multidrug resistance-associated proteins, hence augmenting the effectiveness of oxaliplatin.

Sorafenib has been observed to elicit apoptosis and perhaps mitigate resistance to chemotherapeutic agents, which may be the reason why HAIC combined with sorafenib has a relatively good survival outcome in advanced HCC (75) Lenvatinib, also a multitarget drug, has been shown to inhibit the activity of multidrug resistance-associated transporters and increase the sensitivity of FOLFOX regimen (76). This mechanism may promote the stronger combined antitumor effect of the FOLFOX regimen and Lenvatinib (38, 77). However, it is imperative to acknowledge that triple therapy combined with anti-PD-1 mAb has better tumor response and long-term survival benefit than HAIC combined with targeted therapy. This has led to greater interest in the underlying mechanisms of anti-tumor immune responses generated by combination therapy. It was found that triple therapy can increase the level of CCL 28 and the number of CD 8^+^ and CD 4^+^ T cells in peripheral blood and reduce the level of betacellulin, which inhibits tumor angiogenesis and progression. Furthermore, it was shown that the levels of PD-1 and lenvatinib targets were notably elevated in individuals exhibiting a high expression of CCL28. The subgroup analysis revealed that those in the high CCL28 group had a significantly extended mOS (67). While additional research is required to investigate the cut-off values of CCL28 and betacellulin in a more extensive population, and the assessment of lymphocyte count in the tumor immune microenvironment (TME) was not conducted in this study, it has provided the impetus for further exploration into the underlying processes of combination therapy.

Cytotoxic chemotherapeutic agents (e.g., Gemcitabine, Oxaliplatin, and Cyclophosphamide) can enhance anti-tumor immune responses via many mechanisms. Chemotherapeutic agents induce apoptosis by up-regulating HLA1 and cation-independent M6P receptor (MPR). This upregulation subsequently enhances the sensitivity of cytotoxic T cells, facilitating the infiltration of granzymes, including GrzB, into a broader spectrum of tumor cells (78). GrzB is a member of the granzyme family and is responsible for causing the release of pro-apoptotic mitochondrial mediators into the cytoplasm, so initiating the process of apoptosis (79). Additionally, it interferes with signaling processes and suppresses immunological responses to reinstate immune surveillance functionality (80). The activation of the tumor-specific T cells can be facilitated by blocking the pathway of PD-1/PD-L, which is recognized for inducing immunological escape in tumors through upregulation (81, 82). Following the administration of platinum-based drugs to human colon cancer cells, it was observed that transcription 6 (STAT6) played a crucial role in the regulation of the T-cell inhibitory molecule known as programmed death receptor ligand 2 (PD-L2). The down-regulation of PD-L2 resulted in an increase in the secretion of Th1 cytokines and reversing the Th2-dominant TME, enhancing the recognition of T cells to tumor cells (83). In addition, it has been observed that a pre-administered FP chemotherapy regimen can effectively counteract the immunosuppressive TME and, in conjunction, significantly augment the antitumor efficacy of natural killer (NK) cells. The primary manifestation of this phenomenon is observed in the heightened impact of 5-Fu on the functionality of NK cells, as well as the augmented efficacy of cisplatin in NK cell immunotherapy through the upregulation of UL16 binding protein 2 (ULBP2) (84–86). Specifically, ULBP2 is a ligand that functions as an activation receptor for NK cells, facilitating the cytotoxicity of NK cells towards tumor cells (87). In an experimental mouse model of liver tumors subjected to cisplatin treatment, the expression of the androgen receptor (AR) was suppressed, leading to an augmentation in its degradation. The expression of ULBP2 was up-regulated and the cytotoxicity of NK cells was enhanced due to the down-regulation of AR (86). Cancer cells possess the capacity to augment subpopulations of cells exhibiting pro-tumorigenic characteristics, among which myeloid-derived suppressor cells (MDSCs) are included and impede the immune response against tumors (88, 89). HAIC eliminates cells with suppressive effects on tumor immunity, such as regulatory T cells (Tregs) and MDSCs (90, 91). A retrospective study analysed the immune response of various immune cells in patients treated with FP-HAIC, including tumor-associated antigen (TAA) specific T cells, Treg and MDSCs. It suggested a significant decrease of Treg after anti-tumor treatment (92). Prior research has indicated that the administration of 5-Fu results in the targeted elimination of MDSCs. However, it has been observed that this treatment does not improve the effectiveness of anti-PD-1 mAb through the induction of ICD (93, 94). Hence, solely focusing on the targeting of immunosuppressive cells is insufficient.

The demise of cancer cells within the TME can be categorized into immunogenic and non-immunogenic. In contrast to the process of apoptosis, ICD triggers the activation of pattern recognition receptors (PRRs) in macrophages and dendritic cells (DCs) by the exposure of certain endogenous chemicals known as damage-associated molecular patterns (DAMPs). The antigen presentation process by sensing and activating innate immune cells to T cells is crucial in initiating an anticancer immune response (95). ICD can strongly mimic the immune system and enhance the immune response induced by immunotherapeutic regimens, ultimately contributing to durable protective anti-tumor immunity (96). Cytotoxic drugs can enhance the antigenicity of tumor cells by inducing ICD, while concurrently mitigating the occurrence of unintended immunosuppression in the tumor microenvironment (97). The induction of ICD was shown in colon cancer through *in vitro* investigations, wherein either trifluridine/tipyrimidine (FTD/TPI) or oxaliplatin alone were determined to be responsible for this effect. Additionally, it resulted in the reduction of immunosuppressive tumor-associated macrophages of type 2 (TAM2), reversed the immunological tolerance generated by the tumor, increased the infiltration and activation of cytotoxic CD8^+^ T cells, and enhanced the effectiveness of anti-PD-1 mAb treatment. Nevertheless, it is imperative to note that the induction of ICD in *in vivo* trials necessitates the combination of both medications, so implying that the synergistic utilization of these two agents could potentially enhance the effectiveness of immunotherapy (98). Oxaliplatin can facilitate the initiation of DAMPs, including augmented exposure of CRT, elevated secretion of adenosine triphosphate (ATP), and increased release of high mobility group 1 (HMGB1). Consequently, this process promotes ICD (99). The co-culture system exhibited an augmentation in DCs and activated CD8^+^ T cells, alongside a reduction in Treg cells, whereas such changes were not observed in the cisplatin-treated group (100). DCs assume a pivotal function in the identification of apoptotic cells and the initiation of immune responses. Additionally, they are capable of instigating T cell-mediated anti-tumor response (101). Previous studies have indicated an association has been observed between increased levels of CD8^+^ T cells infiltrating and a favorable prognosis (102, 103). In contrast, Treg cells and B cells expressing PD-1 (often referred to as regulatory B cells) exert inhibitory effects on the aforementioned process (104). In brief, oxaliplatin functions as an inducer of ICD and as a regulator of the TME, thereby facilitating the recruitment of DCs and CD8^+^ T cells to the “tumor bed”. In conjunction with the administration of anti-PD-1 mAb, this approach has the capacity to impede the production of PD-L1 on neoplastic cells, hence facilitating the ability of immune system to identify and engulf these malignant cells. Furthermore, it was discovered that progenitor cells that were depleted and enriched with CD 44^+^PD-1^+^Tim-3^-^ cells exhibited greater susceptibility to anti-PD-1 mAb monoclonal antibody in comparison to terminally depleted CD 8^+^ T cells that were enriched with CD 44^+^PD-1^+^Tim-3^+^ cells (105). The combination of FTD/TPI and oxaliplatin resulted in an elevated proportion of progenitor cells in comparison to terminally depleted CD 8^+^ T cells. Proposing the utilization of this combined therapeutic approach to modulate the T cell phenotype with the aim of augmenting the inhibition of PD-1 actions (98).

**Figure 1:**
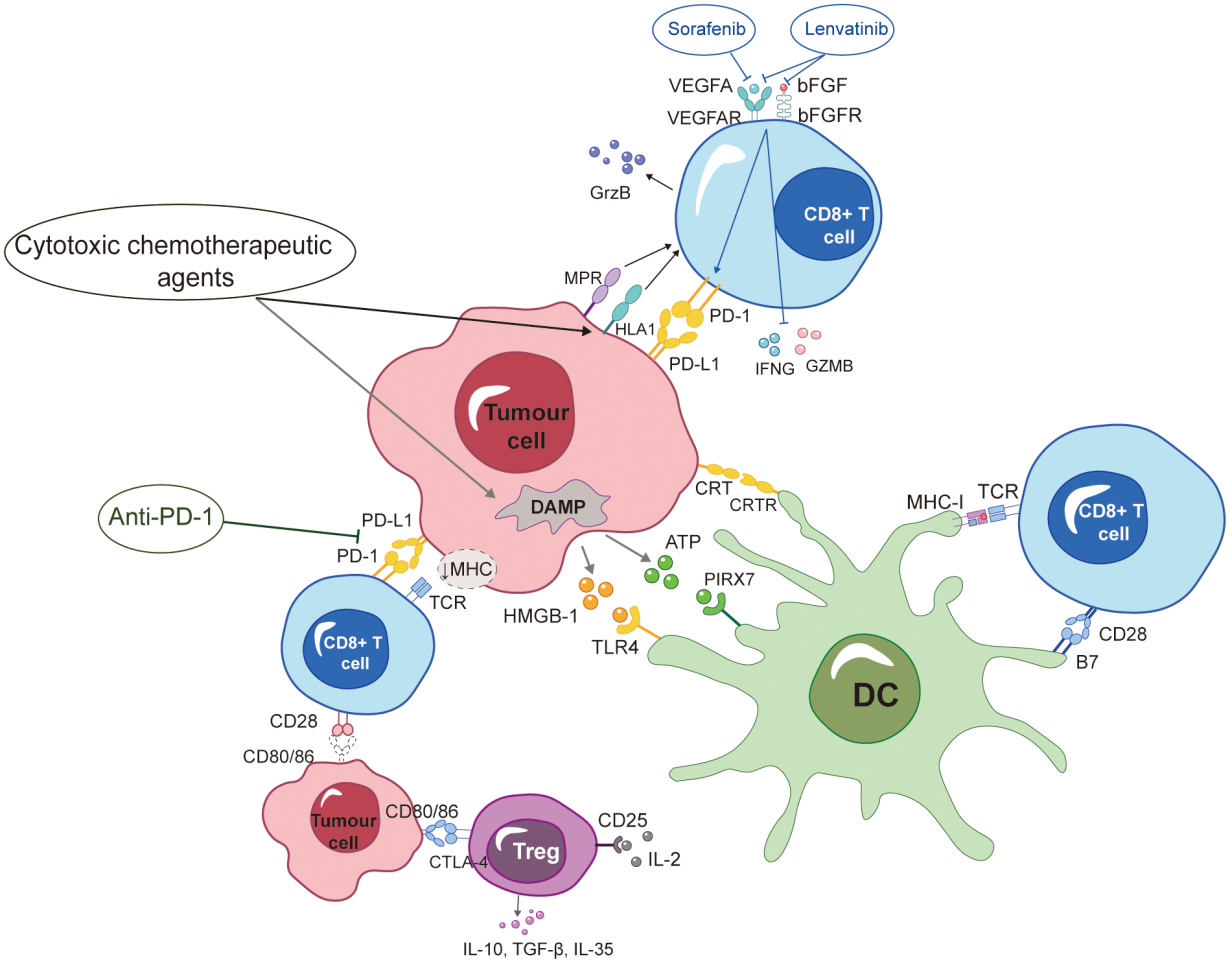
ICD formation is encouraged by HAIC-based chemotherapy drugs, which also affect a variety of immunells and boost anti-PD-1 mAb-induced immune responses against tumors. Additionally, sorafenib and lenvatinib were shown to modulate cytotoxic T-lymphocytes, and immunotherapy had synergistic anti-tumor effects. More specific definitions for the abbreviations are presented at the end of the article.

Lenvatinib appears to have a more potent anti-tumor immune gain effect than sorafenib. Research findings indicated that the expression of PD-1 on T cells was notably increased by vascular endothelial growth factor A (VEGFA) and basic fibroblast growth factor (bFGF). Concurrently, the secretion of interferon gamma (IFNG) and granzyme B (GZMB) was inhibited, and the cytotoxicity of T cells is suppressed. The process was reversed by lenvatinib through the inhibition of two targets. In contrast, sorafenib exclusively targets a single entity. In addition, lenvatinib reduced PD-L1 expression on vascular endothelial cells. This subsequently led to the restoration of T-cell activity, while having no impact on the expression of PD-L1 on HCC tumor cells. Consequently, the sensitivity of tumor cells to PD-1 mAb was preserved (106). Thus, the concurrent administration of PD-1 inhibitors and anti-VEGF medicines has the potential to enhance the anti-tumor response by synergistically modulating the function of effector T cells and normalizing the tumor vasculature inside the TME, thereby converting “cold tumors” into “hot tumors”. In brief, the reported synergistic effect of the anti-PD-1 mAb and HAIC provides a fundamental rationale for the effectiveness of combined therapy in the treatment of advanced HCC (**Figure 1**).

## 6 Discussion

The application of HAIC has demonstrated potential benefits for managing primary intrahepatic lesions in the short term. Hatooka’s study showed that after receiving a course of HAIC, the DCR and ORR were 79.9% and 25.4%, respectively (107). Nevertheless, when sorafenib was exclusively administered as the initial therapeutic intervention for advanced HCC, the DCR ranged from 35.3% to 43%, while the ORR ranged from 2% to 3.3% (43, 108). The Japanese Society of Hepatology (JSH) recommends HAIC as the first-line treatment for advanced HCC patients with large vessel invasion (Vp3 and Vp4), elevated tumor markers, and Child−Pugh B grade liver function (11). While the administration of HAIC as a standalone treatment has demonstrated the ability to regress local tumors at a rapid pace effectively, it may not suffice in adequately controlling the progression of tumors over an extended period in cases with advanced HCC.

We selected representative relevant clinical studies and identified differences in treatment design and efficacy evaluation criteria across the trials described above. Significant disparities were observed in the response evaluations between RECIST 1.1 and mRECIST. The RECIST1.1 criteria use the maximum diameter of the tumor (including the surviving tumor and necrotic areas) to determine the efficacy of the treatment, whereas the mRECIST criteria use the “surviving tumor” to evaluate the efficacy of the treatment (109). Enhanced computed tomography (CT) is frequently employed for evaluating the effectiveness of treatment. The region exhibiting vascular enhancement in arterial enhancement phase corresponds to the active area of HCC, specifically referred to as the target lesion in the mRECIST criteria (110). Due to the potential temporal discrepancy between morphological alterations in images and changes in tumor vascular distribution/activity following antiangiogenic therapy, the evaluation of tumor lesions according to the RECIST1.1 standard does not allow for differentiation between surviving and necrotic lesions post-treatment. No substantial alteration is observed in the overall volume of tumor lesions after local treatment of HCC. However, a notable decrease in the enhancement area and an increase in necrosis area are observed, indicating significant changes in these aspects. The utilisation of mRECIST criteria facilitates the prompt detection of biological responses after therapeutic intervention and identifies the regimen more sensitively (18, 52). Hence, it is advisable to assess the therapeutic efficacy of the combination therapy involving HAIC and anti-PD-1 mAb based on the mRECIST criteria in the context of clinical application.

Several clinicopathological factors can influence the prognosis of patients. Among all HCC patients with vascular invasions, there are 10 to 40% HCC patients were diagnosed with PVTT, of which the median survival time is less than 4 months without effective treatment (111). The present of PVTT not only causes elevated portal pressure and worsening of liver function, but also accelerates the spread of the tumor through the liver, which is closely associated to bad prognosis. There are various treatment modalities for advanced HCC with PVTT, but all have limitations. Transcatheter arterial chemoembolization (TACE) treatment is controversial because of the potential for ischemic liver injury. Systemic chemotherapy provides limited survival benefit. Surgical resection is demanding in terms of tumor site, general condition of the patient and surgical operation (112). HCC patients with PVTT may represent a potentially advantageous cohort for HAIC and anti-PD-1 mAb combination therapy (113). Some studies have shown that after stratification according to the grade of portal vein invasion, the combination therapy group also had a significant survival difference compared with the single drug group (mOS: Vp 3 patients were 16.3 m and 5.5 m, and Vp 4 patients were 13.6 m and 6.5 m; mPFS: Vp 3 patients were 9.9 m and 2.5 m, and Vp 4 patients were 6.8 m and 2.5 m) (52). Furthermore, the question of whether combination therapy benefits the population with extrahepatic metastases remains a subject of controversy. The study conducted by Chen et al. (32) did not demonstrate any significant survival benefit in the group that received combination treatment, probably due to excluding patients with extrahepatic metastases. Other researchers speculated that HCC patients with extrahepatic metastases might possess the demographic traits that would result in a survival advantage in the combination therapy group (18).

The effectiveness and safety of HAIC primarily rely on clinical experience, particularly in East Asian nations. Most research consists of retrospective cohort studies, which are limited in terms of high-quality evidence and large sample sizes. The combination therapy of HAIC and anti-PD-1 mAb similarly encounters the aforementioned predicament. Simultaneously, a considerable proportion of clinical investigations in China predominantly rely on the FOLFOX regimen. While the results demonstrate a more favorable outcome compared to systemic therapy, it is important to note that there exists a considerable prevalence of Hepatitis B virus (HBV) infection among the studied population. A study found that patients with alcoholic cirrhosis or HCV infection were more likely to benefit from the HAIC treatment compared to HBV-infected patients. However, the combination of antiviral medication with lamivudine prolonged the survival of HBV-infected patients, which suggested that antiviral therapy of HBV-infected patients should be standardized throughout the length of HAIC administration (114, 115). Therefore, the applicability of the same treatment paradigm to different regions remains uncertain.

Except the anti-PD-1 mAb, drugs targeting other immune checkpoints include cytotoxic T-lymphocyte associated protein-4 (CTLA-4), programmed cell death-1 (PD-1) and lymphocyte activation gene-3 (LAG-3). The antitumor activity of CD8^+^ T cells is suppressed by CTLA-4 through the upregulation of its expression and its competitive interaction with CD28 for the B7 receptor. Moreover, upregulated CTLA-4 on regulatory T cells (Treg cells) suppresses the activity of dendritic cells (DCs) and CD8^+^ T cells. The aforementioned mechanisms support the possibility of combining anti-PD-1/PD-L1 antibodies with anti-CTLA-4 antibodies as a viable therapeutic approach for HCC. Several studies have been conducted to investigate the efficacy of dual-immunity combinations, which have demonstrated that the use of dual-immunity combinations resulted in improved survival rates and better tumor control outcomes compared to sorafenib monotherapy (116, 117). Nevertheless, there remains a dearth of clinical trials of dual-immunity combinations or anti-CTLA-4 antibodies combined with HAIC.

In this study, we conducted a comprehensive evaluation of the efficacy, safety, and molecular mechanisms underlying the combination of HAIC with anti-PD-1 mAb. HAIC with anti-PD-1 mAb represents a highly advantageous therapeutic approach for advanced HCC. HAIC facilitates the development of TME that is favorable for the effectiveness of anticancer immunotherapy. This is achieved through ICD and the control of immune cell function. Further investigation is required to delve into the molecular processes that contribute to enhancing chemotherapeutic drugs for immunotherapy in the HAIC modality. Additionally, it is imperative to validate these mechanisms through extensive, meticulously planned prospective clinical studies in the future.

## Author contributions

YD: Writing – original draft, Writing – review & editing, Conceptualization, Data curation, Formal Analysis, Investigation. SW: Formal Analysis, Investigation, Writing – review & editing. ZQ: Conceptualization, Methodology, Validation, Writing – review & editing. CZ: Data curation, Validation, Writing – review & editing. YW: Formal Analysis, Investigation, Writing – review & editing. SZ: Investigation, Project administration, Writing – review & editing. WSQ: Writing – review & editing, Supervision. KW: Writing – review & editing, Data curation, Resources. JL: Funding acquisition, Visualization, Writing – review & editing. WWQ: Funding acquisition, Visualization, Writing – review & editing.
